# The Role of Neurofilaments in Diagnosis and Monitoring of Amyotrophic Lateral Sclerosis

**DOI:** 10.3390/jcm15166196

**Published:** 2026-08-10

**Authors:** Anwen Davies, Andrew Bentley, Andras Bikov

**Affiliations:** 1 School of Medical Sciences, University of Manchester, Manchester M14 4PX, UK; anwenhafdavies@gmail.com; 2 Manchester Academic Health Science Centre, Wythenshawe Hospital, Manchester University NHS Foundation Trust, Manchester M23 9LT, UK; andrew.bentley@mft.nhs.uk; 3 Division of Infection, Immunity & Respiratory Medicine, Faculty of Biology, Medicine and Health, University of Manchester, Manchester M14 4PX, UK

**Keywords:** amyotrophic lateral sclerosis, motor neuron disease, neurofilaments, neurofilament light chain, neurofilament heavy chain, respiratory failure, biomarker

## Abstract

**Background**: Amyotrophic lateral sclerosis (ALS), the most common type of motor neurone disease (MND), is a devastating diagnosis that often leads to mortality within 2–5 years of symptom onset. Respiratory failure and aspiration pneumonia both associated with respiratory muscle weakness are the most common causes of death. Difficult to diagnose and devastating in its prognosis, much research has aimed to identify a reliable biomarker to diagnose ALS, prognosticate and improve enrolment into clinical trials to further research efforts. Over the last few decades, neurofilaments (NFs) have emerged as promising biomarkers, especially neurofilament light chain (NFL) and phosphorylated neurofilament heavy chain (pNFH). This review aims to summarise the current evidence for use of NFs as biomarkers in ALS. **Current Evidence**: Higher levels of NFL and pNFH are measured in CSF than in serum, and levels in CSF and serum are correlated. High CSF NFL, serum NFL and CSF pNFH levels could differentiate patients with ALS from healthy controls, other neurological disease, neurodegenerative controls (without MND), other MND subtypes and ALS disease mimics; however, studies reported a high degree of heterogeneity irrespective of which media or NFs have been used. The number of studies examining NFs to predict respiratory failure in patients with ALS is low. **Conclusions and Future Directions**: Despite numerous studies consistently reporting higher NF levels in ALS compared to various controls, their clinical value is limited due to high heterogeneity of the results and inconsistencies in proving its prognostic value. Further understanding the relationship between NF levels and respiratory failure is paramount to improve the quality of life of patients with ALS and increase survival.

## 1. Introduction

### 1.1. Background

Motor neurone disease (MND) refers to a group of rare neurodegenerative disorders [1] that primarily affect the upper motor neurones (UMN), lower motor neurones (LMN), or both [2]. UMN project from higher cortical centres to the brainstem and spinal cord, and LMN refer to anterior horn cells, which project from the brainstem and spinal cord to muscle. Degeneration of UMN and/or LMN is a pathognomonic of MND [3]. Amyotrophic lateral sclerosis (ALS) is the most common type of MND [1] and leads to degeneration of both UMN and LMN [4].

The motor neuron dysfunction leads to symptoms of skeletal (voluntary) muscle weakness. These muscles include those of the upper and lower limbs, the trunk and respiratory muscles. Despite a varied and insidious onset, ALS commonly presents with dysphagia, dysarthria and respiratory dysfunction [4,5]. MND has also been shown to cause cognitive and behavioural changes [2].

The prevalence of ALS in the population is relatively low, presumably due to short life expectancy [6]. The general population’s lifetime risk of developing ALS is approximately 1 in 385 compared to approximately 1 in 70 in those with a first degree relative with ALS [7]. The incidence increases with age, with peak incidence between 70 and 79 years and the mean age of onset between 58 and 63 years. Male sex is a risk factor for development of some ALS subtypes [7,8,9]. Caucasian populations are also at increased risk of developing ALS [10]. Some evidence suggests that smoking and certain occupation-related environmental exposures are associated with ALS [6,11]. Some studies have found an association between a history of playing contact sports and ALS. Early evidence has found that players with repetitive head and cervical spine trauma are at increased risk of ALS, especially those exposed to tobacco [12,13,14].

Alongside environmental factors, genetics play a key role in the heritability and development of ALS [15]. Familial ALS (fALS) is defined as more than one member of a biological family being affected. FALS accounts for between 5% and 10% of all ALS cases [15]. However, this statistic may understate the prevalence of genetic mutations in patients with ALS, with many having inherited genetic mutations from asymptomatic carriers. Four genes have been extensively studied for their implications in the pathophysiology of ALS: C90RF72, FUS, SOD1, and TARDBP [6]. In conclusion, there appears to be a complex interplay between genetics, age, biological sex and environmental factors, with each potentially contributing to disease development and disease phenotype [8].

The diagnosis primarily relies on a thorough history and clinical examination by a specialist in the field, alongside electromyographic studies (EMG). Clinically, ALS can present similarly to ALS-mimicking disorders (ALSmd). ALSmd describe a group of diseases that present similarly to ALS, either through symptoms, clinical presentation or anatomy. These include diseases such as spinobulbar muscular atrophy (Kennedy disease), multiple sclerosis, multifocal motor neuropathy, hereditary spastic paraparesis, and neurologic amyotrophy (Parsonage-Turner syndrome) [16,17,18]. The diagnosis is often delayed due to the low prevalence and rule of more common conditions with similar presentation [19,20]. The diagnosis is commonly made 1 year after symptom onset due to the insidious presentation. On average, ALS leads to mortality within 2–5 years of symptom onset [21]. However, the prognosis ranges from months to years, with some patients surviving decades of disease [22]. Patient survival is primarily limited by development of respiratory failure [21].

Most patients develop type 2 respiratory failure (hypoxaemia with hypercapnia), which eventually leads to death [5]. The decline in respiratory function is partly due to phrenic nerve dysfunction, causing denervation and diaphragm weakness [23]. Decreased respiratory function correlates with decreased quality of life, largely due to the discomfort caused by symptoms. These commonly include dyspnoea, orthopnoea and difficulty in clearing secretions [24]. Studies have also shown that increased dyspnoea positively correlates with the severity of fatigue, which in turn impacts the quality of life of patients with ALS [25].

Early identification of respiratory symptoms, nocturnal hypoventilation and type 2 respiratory failure in patients with ALS guides prescription of medications and devices to alleviate symptoms. At present, patients with ALS and respiratory failure are managed with non-invasive mechanical ventilation (NIV), with some opting for a tracheostomy/invasive mechanical ventilation (IMV). Studies have found that the use of home NIV or IMV prolonged life expectancy in patients with ALS, and there is some evidence to suggest that IMV may prolong life expectancy beyond that afforded to NIV, but this remains less certain [26]. Some evidence suggests that patients who decide to undergo tracheostomy can extend their life expectancy further, on average by 2 years [23]. Use of NIV in patients with ALS, without severe bulbar impairment, has been shown not only to prolong survival time but to preserve quality of life during this period. Earlier use of mechanical ventilation correlated with higher forced vital capacity (FVC), which also correlated with better survival. Earlier identification of patients at risk of hypoventilation may allow for earlier use of mechanical ventilation and better outcomes [26,27].

Methods to monitor respiratory function in ALS include lung function tests, such as FVC and slow vital capacity (SVC); peak cough flow (PCF); Sniff nasal inspiratory pressure (SNIP); maximum inspiratory pressure (MIP); blood gases; and respiratory parameters obtained in sleep studies [24,28,29,30,31]. Identifying a biomarker to predict respiratory failure in ALS patients would facilitate prompt management of alveolar hypoventilation and potentially improve both morbidity and mortality outcomes.

By establishing the mechanisms through which ALS develops and progresses, we may facilitate earlier diagnosis and improve the accuracy of enrolment into clinical trials. At present, trial enrolment is often based on the El-Escorial criteria. However, these criteria lack specificity [22]. The Revised ALS Functional Rating Scale (ALSFRS-R) is widely used to evaluate functional outcomes in ALS clinical trials. It consists of 12 questions assessing gross motor tasks, fine motor tasks, bulbar function and respiratory function. The scale has both high inter-rater and intra-rater reliability, but it remains a subjective score and fails to adjust for symptomatic treatments [32]. Identification of objective biomarkers may inform improved clinical trial inclusion criteria and thereby targeting of therapies.

Research into a reliable biomarker to both identify ALS and monitor progression has been the subject of much research over the last few decades [22]. Neurofilaments (NFs) appear to be a promising diagnostic and prognostic biomarker in ALS [17]. However, despite various studies consistently reporting higher levels of NFs in ALS and a correlation with symptoms, quality of life and life expectancy, these biomarkers are not part of the routine clinical practice.

In this literature review, we will outline the current body of evidence available regarding the diagnostic and prognostic use of NFs in ALS, with an aim to understand the limitations preventing using NFs in clinical practice. We will also examine the potential use of NFs to predict respiratory failure in patients with ALS.

### 1.2. Neurofilaments

NFs are a type of intermediate filaments exclusively found in neurons, with a diameter of approximately 10 nm. They are made up of three major protein subunits: neurofilament light chain (NFL), neurofilament medium chain (NFM) and neurofilament heavy chain (NFH) [17,33]. These subunits are complemented by α-internexin in the central nervous system (CNS) and by peripherin in the peripheral nervous system (PNS). During development, axons are narrow with low amounts of NFs. As axons mature and become myelinated, they accumulate more NFs. NFs are also found in dendrites and neurone cell bodies (perikarya) but in lesser amounts [33,34]. In mature axons, NFs are straight, unbranched and highly organised into parallel arrays. Individual NFs are made up of three parts: a central rod domain, an N-terminal variable domain, and a C-terminal domain. Individual NFs are equally separated by side arms that extend perpendicularly from the filament core [33]. Their presence, gene expression and phosphorylation dictate axonal diameter, myelination, axonal strength, axonal structure and conduction velocity [35]. NFH and NFM are heavily phosphorylated, aiding establishment of a stable axonal cytoskeleton [33,36]. In mature neurons, NFs are mainly found in the axons of motor neurons. NFs form long, thin filamentous networks, which interact with actin filaments and microtubule filaments to form the cytoskeletal network. Together, the three cytoskeletal networks regulate cell migration and thus influence axonal structure [35,37,38].

NFs have been widely investigated in ALS [17]. Axonal damage due to injury, inflammation, ageing and other causes leads to NF release into cerebrospinal fluid (CSF) and then, at relatively lower concentrations, into blood. They are considered reliable markers of chronic and acute neuronal injury and are noted to have a long half-life. Increased levels of NFs, especially NFL and phosphorylated neurofilament heavy chain (pNFH), have been documented in the blood and CSF of patients with ALS. Increased levels of both NFL and pNFH indicate motor neurone damage; however, some evidence suggests that higher NFL levels better correlate with UMN damage, and higher pNFH levels better correlate with LMN damage. It is suggested that pNFH may be able to differentiate between ALS and disease mimics in a more reliable way than NFL. However, NFL has been more extensively researched [16,33,34,36,39,40,41]. Measurements of pNFH have high specificity for neuroaxonal damage and thus may be useful to diagnose ALS [40].

As a diagnostic biomarker, NFL is rather nonspecific, given that it is a marker of neuronal damage [40]. However, ALS pathogenesis involves rapid degeneration of long motor neurone axons, which in turn leads to uniquely high levels of NFL, compared to many other neurodegenerative diseases [16,42].

Phosphorylation of NFs is the most common post-translational modification and plays a crucial role in regulating neuronal function, stability and axonal transport velocity. Phosphorylation of NF subunits promotes proper assembly of NFs and increases resistance to degradation. NFH and pNFH are sometimes used interchangeably in clinical trials due to the understanding that pNFH is more abundant in axons and more stable in CSF, making it an easier biomarker to measure [33,34,35,36,42,43,44]. However, NFH phosphorylation may contribute to ALS progression through the slowing of axonal transport and NFH interaction with other cytoskeletal proteins [20]. Abnormal phosphorylation of NFs can lead to the accumulation of heteroaggregates, seen in ALS. These aggregates are resilient and are resistant to breakdown [36].

## 2. Current Evidence

### 2.1. Neurofilaments in Cerebrospinal Fluid Versus Blood, Analytical Techniques and Stability

Current research proposes two main methods of measuring NFs in a patient population: CSF and blood; the latter analyses performed in both serum and plasma samples [16,17,20,43]. Shahim et al. reported that NFL levels in serum correlated with those in plasma but were ~17% higher and more variable in plasma than serum. There was also a strong correlation between NFL as well as pNFH levels in blood and CSF. However, the correlation between blood and CSF levels for NFL was stronger than for pNFH [17]. These results were supported by Xu et al. [43].

However, different assay techniques were used to measure NFs in blood and CSF in different studies. These methods included enzyme-linked immunoassay (ELISA), electrochemiluminescence-based immune assay (ECL), chemiluminescence enzyme immunoassay (CLEIA) and single molecule array (SIMOA). The differences in sensitivity of these methods may lead to discrepancies in study findings and greater heterogeneity within studies. Some, but not all, meta-analyses acknowledge the different assay techniques used and account for the differences [16,17,20,42,43]. A meta-analysis by Sferruzza et al. found no difference between SIMOA and ELISA for CSF but reported that SIMOA may be more effective in differentiating between NFL blood levels in patients with ALS compared with controls [42]. The same study also found that NFL in CSF had higher diagnostic accuracy compared to blood when comparing the area under the summary receiver operating characteristic curves (AUC) (AUC 0.90 and 0.78 respectively), with further analysis showing higher levels of false positives in blood compared to CSF samples [42].

A longitudinal study by Gaiani et al. found no significant effect of CSF storage time and NFL levels, suggesting its stability in stored samples over time. Clinically, this is important to allow for practical implementation of CSF NFL measurements [45].

There is conflicting evidence to suggest that NF levels remain stable over time. Some studies suggest that levels vary over time and are thus potentially useful in monitoring disease progression. It is thought that total levels of NFs decrease over time and towards the end of disease due to motor neurone destruction having already taken place and no longer releasing NFs [45].

### 2.2. Amyotrophic Lateral Sclerosis Versus Non-Neurodegenerative Controls

Several studies have found that measurement of NFL can distinguish patients with ALS from controls. A meta-analysis conducted by Agah et al. found that CSF levels of NFL were significantly higher in those with ALS compared to controls (patients without ALS) and neurologically healthy controls (NHCs) [20]. Another meta-analysis conducted by Shahim et al. found that CSF NFL showed high sensitivity in distinguishing patients with ALS from controls. The same study showed high specificity (0.90 [95% CI = 0.83, 0.94]) and showed good overall performance. However, the results showed an intermediate-high level of heterogeneity (I^2^ = 83.7%) between studies, reducing reliability of the results. On further inspection, most included studies had high levels of sensitivity (>0.80), and all studies included had a sensitivity >0.75. Specificity varied more, with most results showing a specificity >0.80 and one outlying result of 0.56 [17]. Verde et al. also conducted a meta-analysis that found significantly higher CSF NFL levels in ALS patients compared to NHCs, with an intermediate-high level of heterogeneity (I^2^ = 84%). After age adjustment, the difference did not significantly change [16]. Sferruzza et al. also found that CSF NFL levels were significantly higher in ALS compared to NHCs, with high heterogeneity (I^2^ = 95%) [42]. Xu et al. found significantly higher levels of CSF NFL in ALS patients compared to controls, with intermediate heterogeneity (I^2^ = 82.5%) [43]. A longitudinal study by Gaiani et al. found that CSF NFL was able to reliably differentiate between ALS and controls, noting higher concentrations in patients with ALS [45]. The results of the meta-analyses and individual studies are summarised in Table 1.

The meta-analysis by Agah et al. found significantly raised NFL in blood samples of patients with ALS compared to controls and NHCs [20]. Verde et al. found similar results comparing ALS patients with NHCs, albeit with high heterogeneity (I^2^ = 87%). Age adjustment increased the mean difference between ALS and NHC [16]. Sferruzza et al. concluded that blood NFL was significantly higher in ALS compared to NHCs, with lower heterogeneity (I^2^ = 82%) [42]. Xu et al. found significantly elevated levels of NFL in ALS patient blood compared to controls, with low levels of heterogeneity (I^2^ = 20.4%). One study used ECL; all others used ELISA [43].

The meta-analysis by Agah et al. found that both CSF and blood NFL levels were significantly higher in those with ALS compared to non-neurodegenerative controls (NNCs). The latter group included patients with neurological conditions not considered to be neurodegenerative, such as headache, idiopathic intracranial hypertension and inflammatory polyneuropathies [20]. The results of the meta-analyses and individual studies pertaining to NFL in blood are summarised in Table 2.

Measurements of pNFH in CSF were also highly sensitive and specific for differentiating between patients with ALS and controls. The meta-analysis by Shahim et al. noted a high pooled sensitivity (0.84 [95% CI = 0.79, 0.88]) and specificity (0.83 [95% CI = 0.77, 0.89]). Although the results for CSF pNFH showed lower heterogeneity compared with CSF NFL (I^2^ = 69.6% and I^2^ = 83.7% respectively), they also showed lower levels of sensitivity and specificity compared to NFL [17].

Xu et al. reported significantly higher levels of pNFH in ALS patients’ CSF compared with controls, with intermediate-high heterogeneity (I^2^ = 84.7%). The same study found no difference between pNFH levels in the blood of ALS patients compared with controls, with intermediate-high heterogeneity (I^2^ = 87.5%) [43]. A summary of both meta-analyses and individual studies evaluating the potential use of pNFH as a biomarker to differentiate between patients with ALS and NHCs can be found in Table 3 and Table 4.

### 2.3. Amyotrophic Lateral Sclerosis Versus Other Neurodegenerative Diseases

A meta-analysis conducted by Agah et al. found that CSF levels of NFL were significantly higher in those with ALS compared to neurodegenerative controls (NCs). NCs included patients with conditions such as Parkinson’s disease (PD), frontotemporal lobar degeneration (FTLD) and Alzheimer’s disease (AD) [20]. CSF levels of NFL were significantly higher in those with ALS compared to FTLD and AD [20]. A longitudinal study by Gaiani et al. found that CSF NFL was able to reliably differentiate between ALS and non-ALS frontotemporal dementia (FTD), noting higher concentrations in patients with ALS compared to patients with other FTD [45].

Sferruzza et al. concluded that CSF NFL levels were significantly higher in ALS compared to other neurological diseases (OND), with high heterogeneity (I^2^ = 94%). Blood results were not significantly different, with even higher heterogeneity (I^2^ = 98%) [42].

Verde et al. found higher concentrations of NFL in the CSF of patients with ALS compared to OND but with high heterogeneity between studies (I^2^ = 94%). Similar results were found when comparing levels of NFL in the blood of patients with ALS compared to ONDs, with high heterogeneity (I^2^ = 95%) [16].

Xu et al. found that NFL levels in CSF of patients with ALS were significantly increased compared to OND, with high heterogeneity (I^2^ = 94.3%). The same study found no significant difference between CSF pNFH levels in patients with ALS compared with OND with CNS involvement, with very high heterogeneity (I^2^ = 95.936%) [43].

A meta-analysis conducted by Agah et al. found that CSF levels of NFL were significantly higher in those with ALS compared to ALSmd [20]. A study by Verde et al. similarly found raised CSF NFL levels in ALS patients compared to ALSmd, with intermediate heterogeneity (I^2^ = 62%) [16]. Sferruzza et al. concluded that CSF NFL was significantly higher in ALS compared to ALSmd, with intermediate heterogeneity (I^2^ = 71%) [42]. Xu et al. found that CSF levels of NFL were significantly lower in ALSmd compared to ALS, with very low heterogeneity (I^2^ = 0%), likely due to only including two studies [43].

Comparable results were found when measuring NFL levels in CSF. The meta-analysis by Shahim et al. concluded that CSF NFL showed high sensitivity (0.87 [95% CI = 0.83, 0.89]) and high specificity (0.84 [95% CI = 0.77, 0.88]) in distinguishing ALS patients from ALSmd. The study also found that CSF NFL had high diagnostic accuracy, with a summary receiver operating characteristic (SROC) curve of 0.92 [17]. A longitudinal study by Gaiani et al. found that CSF NFL was able to reliably differentiate between ALS and ALSmd, noting higher concentrations in patients with ALS compared to ALSmd [45].

Behzadi et al. found significantly increased CSF NFL in patients with ALS compared to neuropathies and myelopathies and patients with myelopathies alone [46]. Table 5 summarises the results of studies evaluating the use of CSF NFL as a biomarker to differentiate between patients with ALS and ONDs.

The results for blood samples were similar, finding significantly raised NFL in ALS patient blood compared to NCs, FTLD and AD [20]. Behzadi et al. also found significantly increased plasma NFL in patients with ALS compared to neuropathies and myelopathies and patients with myelopathies alone [46].

Agah et al. found significantly raised NFL in blood in patients with ALS compared to ALSmd [20]. Verde et al. found similar results, with very low heterogeneity (I^2^ = 0%), potentially due to the low number of included studies [16]. Sferruzza et al. found that blood NFL was significantly higher in ALS compared to ALSmd, with high heterogeneity (I^2^ = 90%) [42]. A meta-analysis by Shahim et al. also looked at NFL levels in blood to distinguish between ALS and ALSmd. It showed high sensitivity (0.92 [95% CI = 0.89, 0.94] and specificity (0.90 [95% CI = 0.83, 0.94). The study also found that blood NFL had high diagnostic accuracy, with an SROC curve of 0.95 [17].

Shahim et al. concluded that NFL measured in serum, plasma or CSF was able to distinguish between ALS, ALSmd and controls. Similar results were seen for pNFH in both CSF and serum, but results had increased heterogeneity [17]. Table 6 summarises the results of studies evaluating the use of NFL measured in blood as a biomarker to differentiate between patients with ALS and ONDs.

Shahim et al. found that CSF measurements of pNFH were highly sensitive and specific in distinguishing patients with ALS from patients with ALSmd (0.84 [95% CI = 0.78, 0.88] and 0.86 [95% CI, 0.76 to 0.92] respectively). The SROC curve was 0.91, and results showed intermediate heterogeneity (I^2^ = 73.3%) [17]. Xu et al. found significantly higher levels of pNFH in ALS patients’ CSF compared with ALSmd, with intermediate heterogeneity (I^2^ = 68.8%) [43]. Table 7 summarises the results of studies evaluating the use of CSF pNFH as a biomarker to differentiate between patients with ALS and ONDs.

### 2.4. Amyotrophic Lateral Sclerosis Versus Other Motor Neuron Disease Subtypes

In the meta-analysis by Agah et al., CSF NFL levels were able to differentiate between those with ALS and other MND subtypes. Blood and CSF NFL levels were higher in those with ALS compared to other MND subtypes [20].

A longitudinal study by Gaiani et al. looked at the difference in CSF NFL levels in patients with typical ALS compared to other MND subtypes. Significantly higher levels were found in patients with typical ALS compared to flail leg syndrome, flail arm syndrome, and progressive muscular atrophy. However, no difference was found between levels found in typical ALS compared to progressive bulbar palsy or UMN-dominant ALS [45].

A 2006 study by Brettchneider et al. found that levels of pNFH in CSF were twice as high in patients with predominantly UMN ALS compared to those with predominantly LMN ALS. Evidence of increased CSF NFH concentrations was seen in patients with clinical evidence of UMN symptoms, as well as patients with neurophysiological evidence to corroborate this [44].

A study by Behzadi et al. also looked at the ability of NFs to differentiate between ALS and other MND subtypes. The study found significantly elevated levels of CSF pNFH and plasma NFL in patients with ALS compared to other MND subtypes. However, the results for CSF NFL were not statistically significant. On further subgroup analysis, significantly higher plasma concentrations of NFL were found in patients with bulbar onset ALS compared to spinal onset ALS. However, these results need to be considered with caution as they were not replicated by other studies. When comparing patients with ALS of truncal onset to patients with ALS of bulbar onset or comparing patients with truncal onset ALS to patients with spinal onset ALS, the difference was not significant. There were also some differences noted when comparing ALS patients with different genetic mutations. Patients with ALS associated with C9orf72HRE mutation had significantly increased plasma concentration of NFL compared to patients with ALS associated with SOD1 mutation. However, the difference between patients with C9orf72HRE or SOD1 mutations and patients with ALS without a known genetic mutation was not significant. There was no difference between CSF concentrations of either NFL or pNFH in patients with genetic mutations (SOD1, C9orf72HRE) compared to patients without [46]. The evidence for NF level use to discriminate between non-genetic ALS and genetic forms of ALS is limited; however, it is important that genetic variations do not impair the diagnostic utility of NFs [46].

### 2.5. Relationship to Disease Progression in Amyotrophic Lateral Sclerosis

The meta-analysis by Shahim et al. found that NFL levels were related to disease progression. Higher levels of NFL in CSF and blood corresponded with accelerated disease progression. Comparable results were seen for pNFH in CSF and serum. All results show low levels of heterogeneity between studies and a weak direct relationship between higher NF levels and increased disease progression [17]. Xu et al. found a moderately inverse correlation between CSF pNFH levels and disease duration, with intermediate heterogeneity (I^2^ = 40.1%). A similar correlation was seen when analysing CSF NFL levels with intermediate heterogeneity (I^2^ = 54.0%). These results suggest that increased NF levels correlate with more aggressive disease and shorter life expectancy [43]. A longitudinal study by Gaiani et al. found that higher levels of CSF NFL inversely correlated with disease duration and thus suggested a shortened life expectancy [45]. The meta-analysis by Shahim et al. found that higher baseline levels of blood NFL correlated with shorter survival. Multivariate analyses also confirmed this for CSF and blood NFL as well as CSF and serum pNFH [17]. Behzadi et al. also confirmed that increased concentrations of CSF NFL, CSF pNFH and plasma NFL had an inverse correlation with survival of patients with ALS. When comparing all three biomarkers in terms of estimating prognosis, receiver operating characteristics showed the largest area under the curve for plasma NFL. Both CSF NFL and CSF pNFH levels significantly correlated with disease progression rate [46].

### 2.6. Relationship to Symptoms in Amyotrophic Lateral Sclerosis

Higher concentrations of NFL and pNFH both in blood and CSF were related to lower ALSFRS-R scores [17,43]. However, the relationship for CSF NFL was not significant in other studies [32,43]. The longitudinal study by Gaiani et al. found that higher levels of CSF NFL at baseline correlated with an accelerated decrease in ALSFR-R scores. The same study found an inverse correlation between NFL levels and serial calculations of ALSFRS-R, measured at baseline, 6 months, 12 months, 24 months and 36 months [45]. Behzadi et al. also found that NFL plasma and CSF concentrations inversely correlated with ALSFRS-R and directly related to disease progression rate. However, the results for CSF pNFH were not statistically significant [46].

### 2.7. Amyotrophic Lateral Sclerosis Pre- and Post-Treatment

Xu et al. found no significant difference in blood pNFH or NFL levels of ALS patients treated with Riluzole and those left untreated [43]. A study by Salomon-Zimri et al. looked at the effect of using a combination of ciprofloxacin and celecoxib in patients with ALS on disease progression. Both serum NFL and pNFH levels remained unchanged over time [47]. A randomised, double-blind, placebo-controlled trial evaluated the effect of rapamycin use in patients with ALS compared to placebo. The study found that serum NFL, serum pNFH, CSF NFL and CSF pNFH levels decreased in placebo patients, but the levels were found to remain unchanged in patients taking rapamycin [48].

### 2.8. Relationship to Respiratory Failure in Amyotrophic Lateral Sclerosis

Simkins et al. looked at the correlation between plasma NFL and pNFH concentrations and disease progression. They used a large data set taken from a 48-week, randomised double-blind, placebo-controlled clinical trial looking at the effect of Tirasemativ in patients with ALS. No correlation between baseline SVC and baseline plasma NFL concentrations was found, but increased time in the trial correlated with a stronger correlation between NFL levels and SVC. A weak inverse correlation between baseline serum pNFH ad SVC was found [49].

A retrospective observational study by Koch et al. explored the relationship between serum levels of Cardiac Troponin T and NFL as well as respiratory function. Increased levels of Cardiac Troponin T correlated with a decrease in respiratory function (SVC and FVC). However, despite finding increased serum levels of NFL in some patients with ALS, the levels remained unchanged despite a decrease in respiratory function [50].

In obstructive sleep apnoea, serum NFL levels correlated with the degree of overnight hypoxia, and lower levels were reported in treated patients. Although the study did not investigate ALS, the results suggest that improvement of overnight hypoxia may relate to more favourable NFL levels [51]. These results may partly explain the high heterogeneity of the results, as overnight hypoxia has not been systematically evaluated in studies assessing NFs in ALS.

A retrospective study by Verde et al. explored the relationship between serum levels of NFL and various phenotypes in ALS, including markers of respiratory function. Markers of respiratory function included arterial blood gas measurements, FVC, and nocturnal polysomnography. The authors concluded that there was no correlation between markers of respiratory function and serum NFL. However, the study did find an inverse relationship between serum NFL levels and survival. It also concluded that levels of serum NFL were able to differentiate between patients with ALS and NHCs [52].

## 3. Discussion

This review paper has several clinically relevant conclusions. First, NFs are measurable in both CSF and blood samples [16,17,20,43], and the concentrations correlate well in the two media [17,43]. Whilst the measurement of NFs in blood proves a less invasive method of sample collection [16], both NFL and pNFH levels are higher in CSF compared with blood, likely due to neurone direct contact with CSF [17,43]. Reassuringly, advancements in technology allow reliable measurement of NFs in blood, albeit at lower total levels [16]. Importantly, different assay techniques have been used to measure levels of NFs in CSF and blood [17,20,42]. Whilst the selection of assay technique has no effect on study results when looking at NFL levels in CSF, SIMOA seems to be more sensitive to detect levels of NFL in blood [42]. SIMOA has the ability to measure concentrations in femtolitres, whilst the most sensitive ELISA is able to detect concentrations in the order of ten nanolitres [53]. Higher total levels of NFs in CSF compared with blood may explain why ELISA appears to be equally sensitive to SIMOA [16,42,53]. Nevertheless, the correlation between blood and CSF NFL levels gives further reasoning to use blood samples to measure NF levels in future clinical trials [17,43].

Second, NF levels were higher in ALS compared to non-neurodegenerative disease, neurodegenerative diseases and ALSmd. Importantly, the differences were present irrespective of the age of participants [16]. The diagnosis of ALS is made, on average, 1 year after symptom onset due to insidious presentation, varied presenting symptoms and lack of an objective diagnostic biomarker. It relies on experienced clinicians to make a diagnosis, often after excluding other conditions that can present similarly to ALS [16,17,18,19,20,21]. It is thought that patients with ALS have high levels of NFs released early in their disease course due to the aggressive course and rapid neurone destruction [16,42]. Whilst both NFL and pNFH are able to differentiate between ALS and those without neurodegenerative diseases, pNFH was investigated less frequently [16,17,20,42,43,45,46]. It is hypothesised that pNFH is more specific for neuroaxonal damage and is released in higher concentrations than NFL, suggesting it is potentially more specific than NFL. The use of a sensitive assay reduces the likelihood of false-negative results for NF levels by allowing the measurement of very small concentrations more reliably [16,17,20,33,34,36,39,40,41,42]. The measurement of NFL in blood was also consistently found to be differentiating between patients with ALS and controls [16,20,43,46,50,52]. However, the evidence base was smaller than that of CSF NFL concentrations. All studies using CSF NFL as a biomarker were able to differentiate between patients with ALS and other neurogenerative diseases [16,17,20,42,43,45,46]. However, the study by Gaiani et al. found that there was overlap between ALS and FTD in CSF NFL levels, which suggests that NF levels alone may not be sufficient to reliably diagnose ALS at lower levels. Although the study used a less sensitive ELISA assay, it is unlikely to have affected the CSF results or led to overlapping data. A higher cutoff, alongside clinical examination, may be necessary for accurate diagnosis [45]. Studies exploring the utility of blood NFL as a biomarker to differentiate between ALS and NCs; ALS and FTLD; ALS and AD; ALS and neuropathies; and myelopathies ALS and ALSmd were promising and found higher concentrations in patients with ALS compared to other groups [16,17,20,42,46]. Fewer studies explored the diagnostic use of CSF pNFH. One study did not find a significant difference in CSF pNFH between ALS and OND [43]. Two studies found higher concentrations in patients with ALS compared to ALSmd [17,43]. One study found significantly raised concentrations of pNFH in the CSF of patients with ALS compared to neuropathies and myelopathies and compared to myelopathies alone [46]. It is important to notice the high heterogeneity of the NF results in ALS contributing to limited clinical value of NFs as diagnostic biomarkers. The heterogeneity can be explained by disease activity, temporal decline of the functioning neurons and analytical techniques. It is also noticeable that neurofilaments correlate with disease activity of neurodegenerative diseases [54]. Therefore, cross-sectional comparison studies between ALS and various control groups could be misleading.

Third, there may be differences in NFL levels between ALS subtypes. Patients with bulbar onset ALS had higher CSF levels compared to spinal onset ALS, but there was no difference between levels in patients with spinal or bulbar onset ALS and truncal onset ALS [20,46]. One study found significantly elevated concentrations of pNFH in the CSF of patients with UMN-predominant ALS compared to LMN-predominant ALS. However, another study did not find a difference between the two [44,45]. Corticospinal tract neurons are thought to have high concentrations of NFs, and UMN destruction is thought to cause breakdown of large-calibre axons. This may explain why UMN involvement could lead to proportionally higher CSF NF concentrations. UMN-predominant ALS is thought to have a better prognosis, as opposed to classical ALS (simultaneous UMN and LMN degeneration). Further clarification of this relationship is necessary [44,45,55,56].

Fourth, the ability to predict rapid disease progression may help with end-of-life planning [21,22,23,27]. Increased concentrations of NFL in CSF and blood and pNFH in CSF at the time of measurement were associated with accelerated disease course in patients with ALS [17]. Several studies found an inverse relationship between survival and increased concentrations of CSF and blood NFL, as well as CSF and blood pNFH [17,43,45,46,52]. The life expectancy of a patient with ALS is approximately 2–5 years from symptom onset, but this is highly variable [21,22]. Widespread muscle weakness due to motor neurone destruction eventually leads to respiratory failure, which is noted to be the most common cause of death in patients with ALS, alongside pneumonia secondary to respiratory failure [23,40]. Understanding the correlation between NF levels and survival may indirectly help to understand how NF levels predict respiratory failure, with higher levels predicting earlier respiratory failure. However, the link between NFs and respiratory muscle dysfunction is weak [49] or not significant [50,52].

The emerging evidence for the use of NFL as a prognostic biomarker in ALS is substantial, and the potential use of NF levels in patients with early signs of ALS may help to reduce the time from symptom onset to diagnosis, allowing for earlier implementation of interventions such as respiratory support (NIV, IMV) and potentially improving quality of life and survival [21,23,27]. However, in clinical practice, a blood biomarker that would predict disease onset, rather than respiratory failure, would be desirable. Future research that aims to identify an objective biomarker to predict respiratory failure may aid early intervention and better outcomes for patients. Each disease course is individual, and thus, it may be difficult to predict phrenic nerve dysfunction and the onset of hypoventilation, but clarifying whether there is a relationship between concentrations of NFs in CSF and/or blood and the onset of respiratory failure is essential [23,25,27].

Fifth, NF levels measured both in CSF [17,43,45,46] and blood [17,46] inversely correlated with ALSFRS-R score. Similar results were reported for CSF [17,43,46] and serum [17] pNFH. ALSFRS-R is widely used to monitor disease progression and outcomes in clinical trials. This measurement is subjective and fails to adjust for patients receiving symptomatic treatment. Use of an objective, measurable biomarker such as NFs that directly correlate with disease process in future clinical trials would be favourable [32].

Sixth, NF levels were found unchanged in clinical trials. Initially, this may appear to discredit the use of NF to monitor progress in clinical trials, but the stability of NF levels could be explained by stability of disease, meaning it is a favourable result [46,48,49]. However, one study looking at markers of respiratory function concluded that levels of serum NFL remained unchanged in patients with ALS despite deteriorating respiratory function, which may discredit its use as a marker of disease stability [51]. Early in the ALS disease course, NFs are high due to the high level of motor neurone degeneration. As the disease progresses, motor neurones have already been lost, leading to comparatively lower concentrations of NFs despite disease progression. Further clarification of the relationship between stable levels of NFL and prognosis is necessary [45,47,48]. Current trial enrolment is usually based on the El-Escorial Criteria. These criteria lack specificity. Use of an objective biomarker for future trial enrolment would be preferential [22]. However, as discussed above, unfortunately, NFs lack robust specificity for this role.

## 4. Conclusions

NFs have been largely investigated as objective diagnostic biomarkers in ALS, with evidence emerging to also support their use as prognostic biomarkers. However, their utility to monitor disease in clinical trials has been limited. The largest level of evidence supports use of NFL rather than pNFH, especially in CSF. However, there is emerging evidence to support measurement of NFL in blood. Research exploring the relationship between respiratory failure and NFs is very limited, and future research into this area is warranted.

## Figures and Tables

**Table 1:** Neurofilament light chain levels in the CSF of patients with ALS compared to groups without known neurological disease.

Authors	Year of Study	Type of Study	Number of Participants	Control Group	Results	Significant?	Reference
Agah et al.	2024	Meta analysis	40 studies, 3158 patients with ALS, 4579 control subjects.	Neurologically healthy controls	SMD = 1.62 [95% CI:1.44,1.80]	Yes	[20]
Verde et al.	2024	Meta analysis	56 studies.	Neurologically healthy controls	SMD = 1.72 [95% CI = 1.46, 1.99] Heterogeneity (I^2^ = 84%)	Yes	[16]
Sferruzza et al.	2022	Meta analysis	34 studies, 3122 patients with ALS, 986 controls.	Neurologically healthy controls	SMD = 2.58 [95% CI = 0.86, 4.30] Heterogeneity (I^2^ = 95%)	Yes	[42]
Shahim et al.	2024	Meta analysis	60 studies, 6064 patients with ALS, 2198 controls.	Neurologically healthy controls	Sensitivity 0.91 [95% CI = 0.86, 0.94] Specificity 0.90 [95% CI = 0.83, 0.94]	Yes	[17]
Gaiani et al.	2017	Single centre, retrospective longitudinal study	94 patients with ALS, 44 controls.	Controls (without neurodegenerative disease)	Concentrations in patients with ALS (median 5741.51 pg/mL, interquartile range 2494.26–11,911.51 pg/mL) Concentrations in controls (median 395.53 pg/mL, interquartile range 320.44–804.84 pg/mL)	Yes	[45]
Xu et al.	2016	Meta analysis	6 studies, 463 patients with ALS, 214 healthy controls.	Controls (without neurodegenerative disease)	SMD = 1.627, *p* < 0.0001. Heterogeneity (I^2^ = 82.484%)	Yes	[43]
Agah et al.	2024	Meta analysis	40 studies, 3158 patients with ALS, 4579 control subjects.	Patients without ALS	SMD = 1.46 [95% CI: 1.25, 1.68]	Yes	[20]
Agah et al.	2024	Meta analysis	40 studies, 3158 patients with ALS, 4579 control subjects.	Non-neurodegenerative controls	SMD = 1.22 [CI = 0.94,1.49]	Yes	[20]
Behzadi et al.	2021	Retrospective observational cohort study	234 patients with ALS, 9 controls.	Controls	Elevated in patients with ALS compared to controls (*p* < 0.01) Sensitivity 80.3% Specificity 81.8%	Yes	[46]

**Table 2:** Neurofilament light chain levels in the blood of patients with ALS compared to groups without known neurological disease.

Authors	Year of Study	Type of Study	Number of Participants	Control Group	Results	Significant?	Reference
Agah et al.	2024	Meta analysis	29 studies, 2863 patients with ALS, 3080 controls	Controls	SMD = 1.35 [95% CI = 1.09, 1.60]	Yes	[20]
Agah et al.	2024	Meta analysis	29 studies, 2863 patients with ALS, 3080 controls	Neurologically healthy controls	SMD = 1.50 [95% CI = 1.30, 1.70]	Yes	[20]
Verde et al.	2024	Meta analysis	56 studies	Neurologically healthy controls	SMD = 1.88 [95% CI = 1.32, 2.44] Heterogeneity (I^2^ = 87%)	Yes	[16]
Sferruzza et al.	2022	Meta analysis	18 studies, 1464 patients with ALS, 707 controls.	Neurologically healthy controls	SMD= 1.59 [95% CI = 1.25, 1.92] Heterogeneity (I^2^ = 82%)	Yes	[42]
Xu et al.	2016	Meta analysis	3 studies, 202 patients with ALS, 277 controls	Controls	SMD = 1.448, *p* < 0.0001 Heterogeneity (I^2^ = 20.382%)	Yes	[43]
Behzadi et al.	2021	Retrospective observational cohort study	234 patients with ALS, 9 controls	Controls	Elevated in patients with ALS compared to controls (*p* < 0.01) Sensitivity 76.4% Specificity 83.3%	Yes	[46]
Agah et al.	2024	Meta analysis	29 studies, 2863 patients with ALS, 3080 controls	Non-neurodegenerative controls	SMD = 0.78 [95% CI = 0.32, 1.24]	Yes	[20]

**Table 3:** Phosphorylated neurofilament heavy chain levels in the CSF of patients with ALS compared to groups without known neurological disease.

Authors	Year of Study	Type of Study	Number of Participants	Control Group	Results	Significant?	Reference
Shahim et al.	2024	Meta analysis	60 studies, 6064 patients with ALS, 2198 controls.	Controls	Pooled sensitivity (0.84 [95% CI = 0.79, 0.88]) and specificity (0.83 [95% CI = 0.77, 0.89]) SROC 0.90 Heterogeneity (I^2^ = 69.6%)	Yes	[17]
Xu et al.	2016	Meta analysis	5 studies, 443 patients with ALS, 267 controls.	Controls	SMD = 1.018, *p* < 0.0001 Heterogeneity (I^2^ = 84.696%)	Yes	[43]
Brettchneider et al.	2006	Prospective observational cohort study	69 patients with ALS, 33 controls.	Controls	Patients with ALS 1.7 ng/mL (range 0.0–7.9) Controls 0.3 (range 0.0–1.8) (*p* < 0.001)	Yes	[44]

**Table 4:** Phosphorylated neurofilament heavy chain levels in the blood of patients with ALS compared to groups without known neurological disease.

Authors	Year of Study	Type of Study	Number of Participants	Control Group	Results	Significant?	Reference
Xu et al.	2016	Meta analysis	2 studies, 117 patients with ALS, 78 controls.	Controls	SMD = 1.065 [95% CI = −0.077, 2.207] (*p* = 0.068) Heterogeneity (I^2^ = 87.484%)	No	[43]
Behzadi et al.	2021	Retrospective observational cohort study	234 patients with ALS, 9 controls.	Controls	Elevated in patients with ALS compared to controls (*p* < 0.01) Sensitivity 82.7% Specificity 83.3%	Yes	[46]

**Table 5:** Neurofilament light chain levels in the CSF of patients with ALS compared to groups with known neurological disease.

Authors	Year of Study	Type of Study	Number of Participants	Control Group	Results	Significant?	Reference
Sferruzza et al.	2022	Meta analysis	34 studies, 3122 patients with ALS, 1840 controls.	Other neurological diseases	SMD = 1.31 [95% CI = 0.96, 1.65] Heterogeneity (I^2^ = 94%)	Yes	[42]
Xu et al.	2016	Meta analysis	5 studies, 398 patients with ALS, 405 controls.	Other neurological diseases	SMD = 1.625, *p* = 0.001 High heterogeneity (I^2^ = 94.296%)	Yes	[43]
Agah et al.	2024	Meta analysis	40 studies, 3158 patients with ALS, 4579 control subjects.	Neurodegenerative controls	SMD = 1.29 [95% CI = 0.94, 1.64]	Yes	[20]
Agah et al.	2024	Meta analysis	40 studies, 3158 patients with ALS, 4579 control subjects.	Frontotemporal lobar degeneration	SMD = 0.828 [95% CI = 0.605, 1.052]	Yes	[20]
Gaiani et al.	2017	Single centre, retrospective longitudinal study	94 patients with ALS, 20 controls.	Frontotemporal dementia	Patients with ALS (median 5741.51 pg/mL, interquartile range 2494.26–11,911.51 pg/mL) Patients with FTD (median 2297.50 pg/mL, interquartile range 1529.75–3184.97 pg/mL)	No	[45]
Agah et al.	2024	Meta analysis	40 studies, 3158 patients with ALS, 4579 control subjects.	Alzheimer’s disease	SMD = 1.374 [95% CI = 0.886, 1.863]	Yes	[20]
Agah et al.	2024	Meta analysis	40 studies, 3158 patients with ALS, 4579 control subjects.	Other MND subtypes	SMD = 1.083 [95% CI = 0.664, 1.501]	Yes	[20]
Behzadi et al.	2021	Retrospective observational cohort study	234 patients with ALS, 13 controls.	Other MND subtypes	Elevated in patients with ALS compared to controls (*p* > 0.05) Sensitivity 80.3% Specificity 81.8%	No	[46]
Gaiani et al.	2017	Single centre, retrospective longitudinal study	58 patients with typical ALS, 6 controls.	Flail arm syndrome	Significantly higher log[NFL] levels in patients with typical ALS compared to control Subtype effect F = 7.78, *p* < 0.001	Yes	[45]
Gaiani et al.	2017	Single centre, retrospective longitudinal study	58 patients with typical ALS, 5 controls.	Flail leg syndrome	Significantly higher log[NFL] levels in patients with typical ALS compared to control Subtype effect F = 7.78, *p* < 0.001	Yes	[45]
Gaiani et al.	2017	Single centre, retrospective longitudinal study	58 patients with typical ALS, 9 controls.	Progressive muscular atrophy	Significantly higher log[NFL] levels in patients with typical ALS compared to control Subtype effect F = 7.78, *p* < 0.001	Yes	[45]
Gaiani et al.	2017	Single centre, retrospective longitudinal study	58 patients with typical ALS, 9 controls.	Progressive bulbar palsy	No difference in log[NFL] levels in patients with typical ALS compared to control Subtype effect F = 7.78, *p* < 0.001	Yes	[45]
Gaiani et al.	2017	Single centre, retrospective longitudinal study	58 patients with typical ALS, 7 controls.	UMN dominant ALS	No difference in log[NFL] levels in patients with typical ALS compared to control Subtype effect F = 7.78, *p* < 0.001	Yes	[45]
Agah et al.	2024	Meta analysis	40 studies, 3158 patients with ALS, 4579 control subjects.	ALSmd	SMD = 1.539 [95% CI = 1.307,1.770]	Yes	[20]
Shahim et al.	2024	Meta analysis	60 studies, 6064 patients with ALS, 539 controls.	ALSmd	Sensitivity (0.87 [95% CI = 0.83, 0.89]) Specificity (0.84 [95% CI = 0.77, 0.88])	Yes	[17]
Gaiani et al.	2017	Single centre, retrospective longitudinal study	94 patients with ALS, 18 patients with motor neuropathies.	Motor neuropathies (ALSmd)	Patients with ALS (median 5741.51 pg/mL, interquartile range 2494.26–11,911.51 pg/mL) Motor neuropathies (median 1009.72 pg/mL, interquartile range 596.78–1360.81 pg/mL)	N/A	[45]
Verde et al.	2024	Meta analysis	56 studies.	ALSmd	SMD = 1.18 [95% CI = 0.97, 1.39] Heterogeneity (I^2^ = 62%)	Yes	[16]
Sferruzza et al.	2022	Meta analysis	34 studies, 3122 patients with ALS, 441 controls.	ALSmd	SMD =1.22 [95% CI = 0.95,1.49] Heterogeneity (I^2^ = 71%)	Yes	[42]
Xu et al.	2016	Meta analysis	2 studies, 250 patients with ALS, 99 controls.	ALSmd	SMD = 0.742, *p* < 0.0001 Heterogeneity (I^2^ = 0%)	Yes	[43]
Behzadi et al.	2021	Retrospective observational cohort study	234 patients with ALS, 24 controls.	Neuropathies and myelopathies	Elevated in patients with ALS compared to controls (*p* < 0.01) Sensitivity 80.3% Specificity 81.8%	Yes	[46]
Behzadi et al.	2021	Retrospective observational cohort study	234 patients with ALS, 7 controls.	Myelopathies	Elevated in patients with ALS compared to controls (*p* < 0.01) Sensitivity 80.3% Specificity 81.8%	Yes	[46]

**Table 6:** Neurofilament light chain levels in the blood of patients with ALS compared to groups with known neurological disease.

Authors	Year of Study	Type of Study	Number of Participants	Control Group	Results	Significant?	Reference
Agah et al.	2024	Meta analysis	29 studies, 2863 patients with ALS, 3080 controls.	Other MND subtypes	SMD = 1.118 [95% CI = 0.595, 1.641]	Yes	[20]
Behzadi et al.	2021	Retrospective observational cohort study	234 patients with ALS, 13 controls.	Other MND subtypes	Elevated in patients with ALS compared to controls (*p* < 0.05) Sensitivity 76.4% Specificity 83.3%	Yes	[46]
Sferruzza et al.	2022	Meta analysis	18 studies, 1464 patients with ALS, 216 controls.	Other neurological diseases	SMD = 0.72 [95% CI = −0.39, 1.83] Heterogeneity (I^2^ = 98%)	Yes	[42]
Verde et al.	2024	Meta analysis	56 studies.	Other neurological diseases	SMD = 1.02 [0.59, 1.45] Heterogeneity (I^2^ = 95%)	Yes	[16]
Agah et al.	2024	Meta analysis	29 studies, 2863 patients with ALS, 3080 controls.	Neurological controls	SMD = 1.35 [95% CI = 1.06, 1.65]	Yes	[20]
Agah et al.	2024	Meta analysis	29 studies, 2863 patients with ALS, 3080 controls.	FTLD	SMD = 1.211 [95% CI = 0.813, 1.610	Yes	[20]
Agah et al.	2024	Meta analysis	29 studies, 2863 patients with ALS, 3080 controls.	Alzheimer’s disease	SMD = 1.288 [95% CI = 0.972, 1.603]	Yes	[20]
Behzadi et al.	2021	Retrospective observational cohort study	234 patients with ALS, 24 controls.	Neuropathies and myelopathies	Elevated in patients with ALS compared to controls (*p* < 0.01) Sensitivity 76.4% Specificity 83.3%	Yes	[46]
Behzadi et al.	2021	Retrospective observational cohort study	234 patients with ALS, 7 controls.	Myelopathies	Elevated in patients with ALS compared to controls (*p* < 0.01) Sensitivity 76.4% Specificity 83.3%	Yes	[46]
Agah et al.	2024	Meta analysis	29 studies, 2863 patients with ALS, 3080 controls.	ALSmd	SMD = 1.226 [95% CI = 1.070, 1.383]	Yes	[20]
Verde et al.	2024	Meta analysis	56 studies.	ALSmd	SMD = 1.2 [95% CI = 0.98, 1.41] Heterogeneity (I^2^ = 0%)	Yes	[16]
Sferruzza et al.	2022	Meta analysis	18 studies, 1464 patients with ALS, 329 controls.	ALSmd	SMD = 0.95 [95% CI = 0.42, 1.48] Heterogeneity (I^2^ = 90%)	Yes	[42]
Shahim et al.	2024	Meta analysis	60 studies, 6064 patients with ALS, 539 controls.	ALSmd	Sensitivity (0.92 [95% CI = 0.89, 0.94] Specificity (0.90 [95% CI = 0.83, 0.94) SROC curve of 0.95	Yes	[17]

**Table 7:** Phosphorylated neurofilament heavy chain levels in the CSF of patients with ALS compared to groups with known neurological disease.

Authors	Year of Study	Type of Study	Number of Participants	Control Group	Results	Significant?	Reference
Behzadi et al.	2021	Retrospective observational cohort study	234 patients with ALS, 13 controls.	Other MND subtypes	Elevated in patients with ALS compared to controls (*p* < 0.01) Sensitivity 82.7% Specificity 83.3%	Yes	[46]
Xu et al.	2016	Meta analysis	6 studies, 468 patients with ALS, 329 controls.	Other neurological diseases with CNS involvement	SMD = 0.811 [95% CI = −0.082, 1.705]) (*p* = 0.075) Heterogeneity (I^2^ = 95.936%)	No	[43]
Behzadi et al.	2021	Retrospective observational cohort study	234 patients with ALS, 24 controls.	Neuropathies and myelopathies	Elevated in patients with ALS compared to controls (*p* < 0.01) Sensitivity 82.7% Specificity 83.3%	Yes	[46]
Behzadi et al.	2021	Retrospective observational cohort study	234 patients with ALS, 7 controls.	Myelopathies	Elevated in patients with ALS compared to controls (*p* < 0.01) Sensitivity 82.7% Specificity 83.3%	Yes	[46]
Shahim et al.	2024	Meta analysis	60 studies, 6064 patients with ALS, 539 controls.	ALSmd	Sensitivity 0.84 [95% CI = 0.78, 0.88] Sensitivity 0.86 [95% CI, 0.76 to 0.92] SROC 0.91 Heterogeneity (I^2^ = 73.3%)	Yes	[17]
Xu et al.	2016	Meta analysis	2 studies, 251 patients with ALS, 100 controls.	ALSmd	SMD = 0.796 [95% CI = 0.171, 1.422]) (*p* = 0.013) Heterogeneity (I^2^ = 68.817%)	Yes	[43]
Brettchneider et al.	2006	Prospective observational cohort study	27 patients with UMN dominant ALS, 36 patients with LMN dominant ALS.	UMN dominant vs. LMN dominant ALS	UMN dominant −2.3 ng/mL (range 0.2–7.9) LMN dominant −1.2 ng/mL (range 0.0–1.8) (*p* = 0.02)	Yes	[44]
Brettchneider et al.	2006	Prospective observational cohort study	69 patients with ALS, 73 patients with Alzheimers disease.	Alzheimer’s disease	ALS 1.7 ng/mL (range 0.0–7.9) AD 0.14 (range 0–0.97). *p* < 0.001	Yes	[44]

## Data Availability

No new data were created or analysed in this study. Data sharing is not applicable to this article.

## Abbreviations

The following abbreviations are used in this manuscript:

ALS	Amyotrophic lateral sclerosis
MND	Motor neurone disease
NFs	Neurofilaments
NFL	Neurofilament light chain
pNFH	Phosphorylated neurofilament heavy chain
NFM	Neurofilament medium chain
NFH	Neurofilament heavy chain
UMN	Upper motor neurone
LMN	Lower motor neuron e
fALS	Familial amyotrophic lateral sclerosis
EMG	Electromyographic studies
ALSmd	Amyotrophic lateral sclerosis-mimicking disorders
NIV	Non-invasive ventilation
IMV	Invasive mechanical ventilation
FVC	Forced vital capacity
SVC	Slow vital capacity
PCF	Peak cough flow
SNIP	Sniff nasal inspiratory pressure
MIP	Maximum inspiratory pressure
ALSFRS-R	Revised Amyotrophic Lateral Sclerosis Functional Rating Scale
CNS	Central nervous system
PNS	Peripheral nervous system
CSF	Cerebrospinal fluid
ELISA	Enzyme-linked immunoassay
ECL	Electrochemiluminescence-based immune assay
CLEIA	Chemiluminescence enzyme immunoassay
SIMOA	Single molecule array
AUC	Area under the summary receiver operating characteristic curve
CI	Confidence interval
SMD	Standardised mean difference
NHCs	Neurologically healthy controls
NNCs	Non-neurodegenerative controls
NCs	Neurodegenerative controls
PD	Parkinson’s disease
FTLD	Frontotemporal lobar degeneration
AD	Alzheimer’s disease
FTD	Frontotemporal dementia
OND	Other neurological diseases
SROC	Summary receiver operating characteristic
